# An innovative model of access and triage to reduce waiting in an outpatient epilepsy clinic: an intervention study

**DOI:** 10.1186/s12913-023-09845-2

**Published:** 2023-08-31

**Authors:** Annie K. Lewis, Nicholas F. Taylor, Patrick W. Carney, Xia Li, Katherine E. Harding

**Affiliations:** 1 Eastern Health; Allied Health Clinical Research Office, Level 2, 5 Arnold St, Box Hill, Victoria, 3128 Australia; 2 La Trobe University; School of Allied Health, Health Services and Sport, La Trobe University, Kingsbury Drive, Bundoora, VIC 3086 Australia; 3 Monash University, 21 Chancellors Walk, Clayton, VIC 3800 Australia; 4 The Florey Institute for Neuroscience and Mental Health, Melbourne Brain Centre, Burgundy Street, Heidelberg, VIC 3084 Australia; 5 Department of Mathematical and Physical Sciences, La Trobe University, Kingsbury Drive, Bundoora, VIC 3086 Australia

**Keywords:** Outpatient clinics, Epilepsy, Wait, Access, Appointment, Patient flow

## Abstract

**Background:**

Delayed access to outpatient care may negatively impact on health outcomes. We aimed to evaluate implementation of the Specific Timely Appointments for Triage (STAT) model of access in an epilepsy clinic to reduce a long waitlist and waiting time.

**Methods:**

This study is an intervention study using pre-post comparison and an interrupted time series analysis to measure the effect of implementation of the STAT model to an epilepsy clinic. Data were collected over 28 months to observe the number of patients on the waitlist and the waiting time over three time periods: 12 months prior to implementation of STAT, ten months during implementation and six months post-intervention. STAT combines one-off backlog reduction with responsive scheduling that protects time for new appointments based on historical data. The primary outcomes were the number of patients on the waitlist and the waiting time across the three time periods. Secondary outcomes evaluated pre- and post-intervention changes in number of appointments offered weekly, non-arrival and discharge rates.

**Results:**

A total of 938 patients were offered a first appointment over the study period. The long waitlist was almost eliminated, reducing from 616 during the pre-intervention period to 11 post-intervention (*p* = 0.002), but the hypothesis that waiting time would decrease was not supported. The interrupted time series analysis indicated a temporary increase in waiting time during the implementation period but no significant change in slope or level in the post- compared to the pre-intervention period. Direct comparison of the cohort of patients seen in the pre- and post-intervention periods suggested an increase in median waiting time following the intervention (34 [IQR 25–86] to 46 [IQR 36–61] days (*p* = 0.001)), but the interquartile range reduced indicating less variability in days waited and more timely access for the longest waiters.

**Conclusions:**

The STAT model was implemented in a specialist epilepsy outpatient clinic and reduced a large waitlist. Reductions in the waitlist were achieved with little or no increase in waiting time. The STAT model provides a framework for an alternative way to operate outpatient clinics that can help to ensure that all people referred are offered an appointment in a timely manner.

## Introduction

Early access to outpatient care for people with seizures and epilepsy can lead to earlier diagnosis, commencement of medication or referral for surgery. In turn, timely access to these interventions is recommended for better seizure control, having positive impacts on people’s health, wellbeing and productivity [1]. These crucial interventions are commonly accessed through public outpatient services where a specialist neurologist provides expert diagnosis and initiation of optimal management. People living with epilepsy, as well as those who experience a seizure for the first time, need timely access to specialist neurological care.

Waiting for outpatient care is a well-documented and complex problem experienced in many clinics [2, 3], including in the specialty of epilepsy. There is high demand for epilepsy care based on disease prevalence and timely access is variable and a challenge in many countries [4]. While delayed access impacts individuals [5, 6], it also has flow-on effects resulting in avoidable waste within other parts of the health system [7]. A timely outpatient appointment can reduce preventable emergency department attendance, length of inpatient stay or unnecessary visits to a general practitioner [8]. The problem of waiting is particularly vexed for patients assessed as being of lower priority, who tend to be pushed down the queue to make way for more urgent cases [9, 10]. This occurs despite the possibility of poorer outcomes due to unmet need [11]. Therefore, managing demand for *all* patients, including those deemed lower priority, requires a nuanced approach to waitlist management.

Multi-pronged strategies involving process improvements, alignment of resources and efforts to improve operational efficiency have been associated with effective management of outpatient clinic waitlists [2]. ‘Process improvement’ includes strategies to streamline care pathways, such as use of telehealth, reducing unwanted variability, and eliminating system waste. Strategies under the ‘alignment of resources’ category include rationing, triaging and waitlist auditing. ‘Operational efficiency’ refers to scheduling initiatives based on supply and demand, such as the advanced access model in primary care [12].

A data-driven model of access and triage, STAT (Specific Timely Appointments for Triage), sets out a framework which integrates strategies that have been successful in reducing waiting time [13]. STAT combines a one-off backlog reduction effort to align resources (for example, through auditing or temporary increases in supply), with operational efficiency achieved through ongoing, responsive scheduling that protects time for new appointments based on historical data. Furthermore, STAT provides impetus for process improvements in clinic management. For example, demand can be reduced by refining acceptance and discharge criteria, and efficiency increased by reducing administrative duplication or minimising missed appointments. The STAT model’s structure, assumptions and principles, are supported by evidence synthesising strategies to reduce waiting [2] and align with health policy priorities to improve access to specialist care [14–16].

In multi-disciplinary community and outpatient services, trials of the STAT model have resulted in a 30–40% reduction in waiting time [13, 17, 18]. Further, there is early evidence that the model is being implemented outside the research setting in community services [19]. Reducing waiting in community outpatient settings, where STAT has previously been trialled, is associated with improved patient outcomes [20]. However, waitlists are arguably an even greater problem in specialist medical outpatients [2]. Delays in outpatient epilepsy care adversely impact patient outcomes [21]. A systematic review synthesising results from 35 studies demonstrated that delays in diagnosis, treatment initiation and surgery are associated with unfavourable seizure status in children and adults. Further, prompt access to specialist outpatient neurology care improved quality of life and productivity, and is potentially lifesaving. For children, earlier epilepsy care is associated with improved developmental outcomes [21].

The STAT model may be applicable to specialist medical outpatient clinics, but these services differ from multi-disciplinary community outpatient settings in a range of ways. These include different staffing levels, funding streams, reporting and governance, alignment within the health system and referral volume. However, these different healthcare settings also share characteristics; both favour the use of waitlists to manage demand for non-urgent cases, and use models of care that involve an initial assessment appointment followed by review or treatment appointments. It is common practice in both multi-disciplinary community outpatient and specialist medical outpatient settings to triage referrals to manipulate the order in which patients are offered appointments.

While the STAT model has reduced waiting time in non-medical community outpatient services [13, 17, 18], it remains untested in specialist medical outpatient clinics and previous findings cannot necessarily be generalised. Therefore, this study aimed to evaluate whether the STAT model could be implemented in an epilepsy clinic. It was hypothesised that the numbers of patients on the waitlist and waiting time would be reduced after implementation of STAT.

## Methods

### Design

This study is an intervention study using pre-post comparison and an interrupted time series analysis to measure the effect of implementation of the STAT model to an epilepsy clinic. A protocol has been published [22] and the study is reported here in accordance with the STrengthening the Reporting of OBservational studies in Epidemiology (STROBE) checklist [23].

Routine healthcare data were collected over the study period of 28 months. The study included a 12-month pre-intervention period, from 1 May 2018 to 30 April 2019 where retrospective data were collected. This was followed by a 10-month implementation period and a six-month post-intervention period from 1 March 2020 to 31 August 2020.

Approval for the project was granted by the health service and university research ethics committees (LR19/014). Individual patient informed consent was not sought as the study used routinely collected outcome data. All data were de-identified for analysis.

### Setting

This study was conducted in a specialist medical outpatient epilepsy clinic at an Australian public metropolitan hospital. The clinic ran one afternoon (3.5 h) each week and was staffed by four neurologists specialising in epilepsy. The allocated time for first appointments was 30 min. People were referred following a first suspected seizure or with previously diagnosed epilepsy. The hospital was reimbursed for each appointment by Medicare, the Australian universal healthcare system. Patients did not incur any out-of-pocket expense.

Over the two years prior to the intervention the number on the waitlist had been stable, ranging from 600 to 700 referrals at any given time, with the longest waiter remaining on the list for eight years. This long but stable waitlist suggested that appointment supply had been keeping up with demand, but there was an entrenched backlog and no active plan to manage the large number of waiting patients.

### Participants

Participants were patients of the epilepsy clinic who were either offered a first appointment or were waiting to be allocated a first appointment during the study period.

### Intervention

The Specific Timely Appointments for Triage (STAT) model was applied as described in the STAT Handbook [24]. The STAT model maintains patient flow by protecting appointments for new patients at the rate of demand. It requires a one-off backlog reduction effort at commencement of implementation. Researchers who had previously applied the model in non-medical settings led the implementation, in partnership with the senior epilepsy neurologist. Key stakeholders involved were the director and nurse-unit-manager of specialist clinics, the epilepsy clinic administration officers and the three other neurologists staffing the clinic. Effective full-time equivalent staffing remained stable throughout the study.

Implementation of the STAT model combined a one-off backlog reduction strategy, with a range of changes to maintain balance of required and available new appointments [22]. Demand was calculated using two years of historic data showing number of received referrals, number of referrals rejected or withdrawn, and the fail-to-attend rate. Nine new appointments each week were required to keep up with demand. The existing appointment booking templates were used to calculate and monitor supply of new and follow-up appointments.

A waitlist reduction strategy was developed with stakeholders and commenced with an audit to ascertain the current need of everyone on the waitlist. Patients were removed from the waitlist if their referral was listed as waiting due to an administrative error, the patient had multiple missed appointments, or if they no longer needed the service. Concern for safety of the patients discharged without an appointment was managed by contacting patients, their next of kin, and/or their GPs, and inviting re-referral if required. The senior neurologist provided oversight throughout the audit. Without a waitlist, re-referrals would be booked in promptly. After ascertaining current need, those patients still requiring the service were offered appointments in one-off, temporary extra clinics with a locum neurologist. In addition, some extra appointments were scheduled into staff neurologists’ appointment templates for a limited number of weeks. The waitlist reduction strategy was funded by a modest supporting grant of $10,500AUD. This was spent on a project officer to co-ordinate the strategy and phone patients or their GPs ($5,700 AUD), a nurse assisting with auditing and phone calls ($2,600AUD) and four extra clinics by a locum neurologist ($2,200AUD). The results and further details of the waitlist reduction phase have been reported elsewhere [25].

With the waitlist reduced, and neurologists’ appointment templates matched to demand with nine new appointments available each week, operating the clinic under the STAT model meant that eligible referrals were booked directly into a new appointments without transferring them to a waitlist. To absorb the consistent in-flow of new patients, a range of administrative and clinic process improvements were initiated. These efficiency measures to support ongoing balance in supply and demand were designed to prevent the waitlist returning. On the demand-side, referral criteria were adjusted. Referrals that were more likely to require the skills of an epilepsy specialist were prioritised, such as patients with medication refractory epilepsy. Requests for a second opinion or for review of chronic but stable epilepsy were redirected back to the GP or to private providers. For example, referrals requesting a second opinion or for people living outside the catchment area were redirected. Other measures to increase clinic capacity using existing resources included strict adherence to non-attendance policies and clarification of discharge criteria to manage demand for follow-up appointments.

### Outcome measures

The primary outcome of waiting was measured using both waiting time and number of patients waiting on the waitlist [26]. ‘Waiting time’, defined as the number of days that had elapsed since the time of referral, was collected for each new patient who attended the clinic during the period of the study. The ‘number of patients waiting on the waitlist’ was the number who had been referred but not yet been allocated an appointment. The number of patients on the waitlist was collected on the first business day of each month from commencement of the implementation through to the end of the post-intervention period. Some monthly waitlist counts were available retrospectively from health service reports that had been generated during the pre-intervention period.

Secondary outcomes were the average number of new appointments offered at the clinic each week; rates of attended versus missed appointments (including cancelled or failed to attend); and appointment outcome (classified as rebooked for follow-up versus discharged).

### Data analysis

Analysis was guided by the recommendations of Bernal [27] with simple descriptive comparisons of pre- and post-intervention data followed by interrupted time series analyses of waiting time and number on the waitlist.

The median number of patients on the waitlist each month, and the median waiting time were compared from pre- and post-intervention periods using Mann Whitney U tests. The waiting time interquartile range and 90^th^ percentiles were also described.

Patient characteristics and secondary outcomes from the pre- and post-intervention periods were compared, with Mann Whitney U tests for continuous data to account for data skewness, and Chi squared statistics or odds ratios for nominal data as appropriate. Descriptive statistics and pre-post comparisons were completed in SPSS version 25.

The trend in the number of patients on the waitlist and the median waiting time was estimated with interrupted time series analyses. The number of monthly observations for number on the waitlist was based on the time and data available for the period of study, with 23 data points available. Linear interpolation was used to impute five missing values from the pre-intervention period. There were 28 data points for waiting time (monthly median number of days waited) which was observed for 12 months pre-intervention, ten months during implementation and six months post-intervention. The number of time points was estimated to be sufficient to provide moderate power, given that a strong intervention effect, minimal seasonal effects and even distribution of data points were anticipated [28].

The first interrupted time series model used the Poisson (Quasipoisson) link function where the dependent variable was the number of patients on the waitlist. Missing data were imputed by using linear interpolation. Equidispersion was tested against overdispersion and/or underdispersion to check if Poisson or Quasipoisson link function should be used. For waiting time interrupted time series model, the Gaussian link function was used. Models were tested for autocorrelation. The interrupted time series analyses were performed in R studio version 3.4.3. Statistical significance for parameter estimation was set at α = 5% (*p* ≤ 0.05).

## Results

For the interrupted time series, a total of 938 patients was included in the three time periods pre-intervention (*n* = 376), implementation (*n* = 363) and post-intervention periods (*n* = 199). The sample size was 575 for direct comparison of pre-intervention group (*n* = 376) and post-intervention group (*n* = 199).

### Participant characteristics

The pre- and post-intervention cohorts were similarly distributed in sex and age. The proportion of referrals received from a hospital source rather than community general practitioner was higher post-intervention (79%), compared to pre-intervention (69%) (*p* = 0.007) (Table 1).

**Table 1:** Participant characteristics, pre- and post-intervention

	Pre-intervention (*n* = 376)	Post-intervention (*n* = 199)	Test statistic	*p* value
Sex [n (%)]				
Female	174 (46)	98 (49)	*X*^*2*^(1) = 0.46	0.497
Male	202 (54)	101 (51)		
Age in years				
Median [IQR]	41 [26–58]	44 [26–62]	*U* = 39,855.50	0.197
Referral source [n (%)]				
Hospital	257 (68)	157 (79)	*X*^*2*^ (1) = 7.18	0.007
GP/Community	119 (32)	42 (21)		

*IQR* interquartile range, *X*^*2*^ Chi square, *U* Mann Whitney U test

### Primary outcome: number on the waitlist

Simple pre-post descriptive comparisons of the main outcomes showed the median number of referrals on the waitlist at monthly time points decreased from 616 to 11 (*p* = 0.002) (Table 2).

**Table 2:** Comparison of primary outcomes, pre- and post-intervention

	Pre-intervention	Post-intervention	*Mann Whitney U-Test*
Number on waitlist on first business day of each month			
Median [IQR]	616 [563–755]	11 [7-18]	*p* = 0.002
Time points (n)	5	6	
Waiting time (days)			
(Median [IQR])	34 [25–86]	46 [36–61]	*p* = 0.001

*SD* standard deviation, *IQR* interquartile range

For interrupted time series analysis, the data did not violate assumptions of autocorrelation and partial autocorrelation (Table 3).

**Table 3:** Quasi-Poisson regression model result for number on the waitlist

Predictors	Estimate	StdErr	Lower 95%CI	Upper 95%CI	*P* value
(Intercept) (pre-intervention)	**6.620**	0.104	6.450	6.791	< 0.001
Group					< 0.001
Group 1 (implementation)	**0.720**	0.170	0.440	0.999	< 0.001
Group 2 (post-intervention)	**-3.817**	1.181	-5.759	-1.875	0.004
Time	**-0.038**	0.017	-0.067	-0.009	0.042
Time since period 1	**-0.327**	0.041	-0.395	-0.259	< 0.001
Time since period 2	0.158	0.275	-0.293	0.610	0.571

Pre-intervention: 6.620–0.038*Time

Implementation: **6.620**–0.038*Time + (**0.720**–0.327* Time since implementation)

Post-intervention: **6.620**–0.038*Time + (**–3.817** + 0.158* Time since post-intervention)

**Fig. 1:**
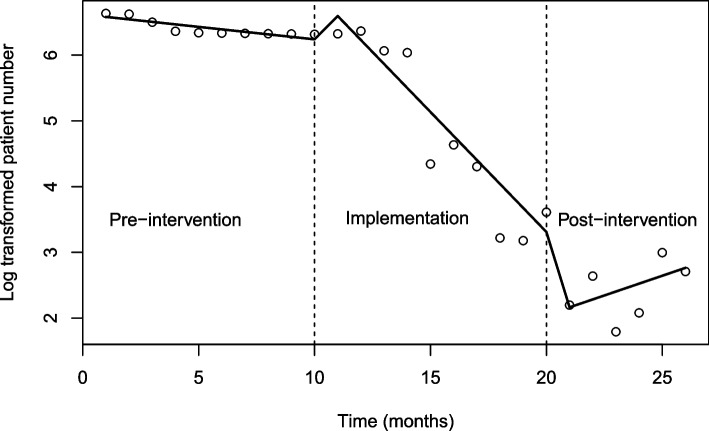
Trend line (slope and level) showing log number of patients on the waitlist during 3 time periods

There was a significant change of level (i.e. the number on the waitlist) between pre- and post-intervention (*p* = 0.004). There was a significant change in slope during the implementation period compared to the pre-intervention period (*p* < 0.001), but no change in slope between pre-intervention and post-intervention (Fig. 1).

### Primary outcome: waiting time

**Fig. 2:**
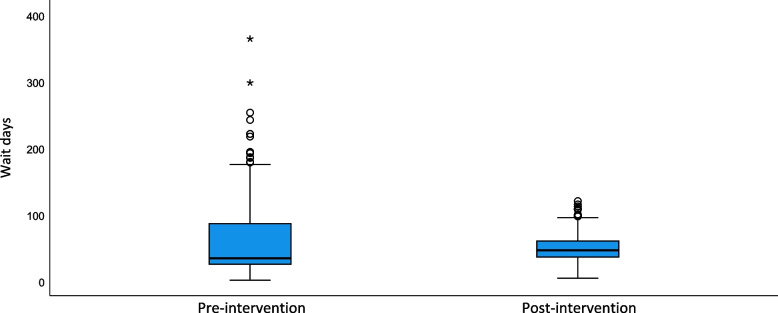
Pre-post comparison of waiting time showing mean and interquartile range

In a pre-post comparison, the median waiting time increased from 34 to 46 days (*p* = 0.001) but reduced variability was observed as indicated by the narrowed interquartile range (25–86 days pre-intervention to 36–61 days post-intervention) (Table 2 and Fig. 2). The 90^th^ percentile of waiting time reduced from 129 days pre-intervention to 87 days post-intervention.

**Fig. 3:**
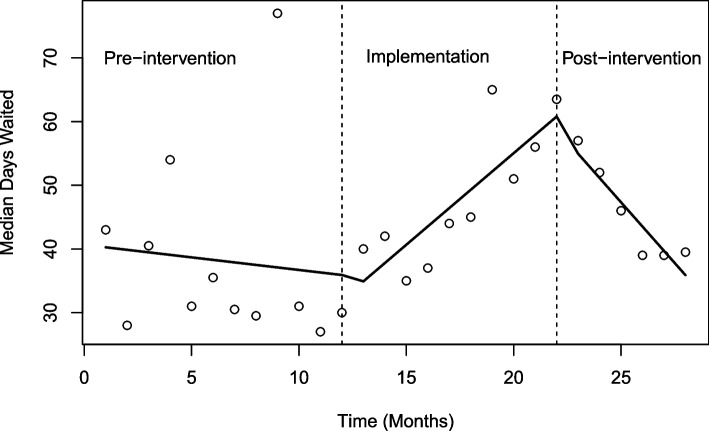
Interrupted time series analysis, median monthly waiting time. Each dot represents the average wait time each month of the series

The interrupted time series analysis indicated that there was no change between pre- and post-intervention in level (i.e. the median waiting time) or slope (Table 4, Fig. 3). The median monthly waiting time slope increased during the implementation period (*p* = 0.043).

**Table 4:** Linear regression model results for waiting time

Predictors	Estimates	CI	*P* value
(Intercept) (pre-intervention)	40.65	26.57 – 54.74	** < 0**.**001**
Group 1 (implementation)	-3.88	-23.85 – 16.09	0.691
Group 2 (post-intervention)	26.81	-10.31 – 63.92	0.148
Time	-0.40	-2.31 – 1.52	0.673
Time since period 1	3.27	0.11 – 6.43	**0**.**043**
Time since period 2	-3.42	-9.21 – 2.38	0.234

R^2^ / R^2^ adjusted

0.362 / 0.217

### Secondary outcomes

There was no change in the number of new appointments provided by the clinic each week. Compared with the pre-intervention period, patients in the post-intervention group were less likely to miss their appointment (OR = 0.57, 95%CI 0.40 to 0.83), and they were less likely to be rebooked for a follow-up appointment, rather than discharged (OR = 0.64, 95%CI 0.42 to 0.99) (Table 5).

**Table 5:** Comparison of secondary outcomes, pre- and post-intervention

	**Pre-intervention (12 months)**	**Post-intervention (6 months)**	**Mean difference or odds ratio (95% CI)**
Number of new appointments scheduled per week			
Mean (SD)	9.7 (5.3)	10.5 (3.6)	-0.81 (-3.1 to 1.5)^a^
Attendance at first appointment [n (%)]			
Attended	221 (59)	142 (71)	0.57 (0.40 to 0.83)^b^
Missed	155 (41)	57 (29)	
First appointment outcome [n (%)]			
Rebooked	315 (84)	153 (77)	0.64 (0.42 to 0.99)^b^
Discharged	61 (16)	46 (23)	

*SD* standard deviation

^a^Independent samples t-test

^b^Odds ratio

There was no evidence to suggest the development of a secondary waitlist for follow-up appointments. The number of days to next available review appointment decreased from a median of 71 [IQR 55–81] during the implementation period to 33 [IQR 24–41] days (*p* = 0.004) in the post-intervention period.

## Discussion

Implementation of the STAT model in an epilepsy clinic resulted in a long waitlist being almost eliminated. The hypothesis that waiting time would decrease, as seen in previous studies [13, 17, 18] was not supported. The interrupted time series analysis indicated no significant change in the median number of days waited, and direct comparison of the cohort of patients seen in the pre- and post-intervention periods suggested an increase in median waiting time following the intervention.

When planning this study, our original hypothesis made an incorrect assumption about the problem of waiting time in this setting. The concept of waiting can be approached in two ways: the number of people on the waiting list, and the time waited for each patient to receive an appointment. The large number of referrals on the waitlist was clear to all involved, and the catalyst to implement and evaluate the STAT model in the epilepsy clinic. It was assumed that a long waitlist would also be associated with long waiting times, but data to confirm this was not easily accessible and reflected a broader problem in accuracy of health services data [29].

During the course of the study, it became apparent that the pre-intervention waiting time, for those who received an appointment, was more reasonable than expected, at a median of 34 days, albeit with considerable variation (IQR 25–86). Therefore, the true problem in this clinic was not overall waiting time but inequity in service provision; some patients received very timely services, some experienced delays, and others were placed on a waiting list indefinitely and never offered a service.

During the waitlist reduction phase clinical risk related to discharging patients without an appointment was mitigated through careful auditing and communication with the patient and GP. We were reassured by a study from the United Kingdom that followed up patients who were discharged from an epilepsy clinic without receiving an appointment and found that 92% did not require the service; they had accessed care elsewhere or had had no further seizures [30]. Furthermore, it was felt that a process of actively approaching patients and discharging them back to their GPs for review was preferable to leaving some patients on a waitlist without a reasonable prospect for timely review by a neurologist.

The lack of change or small observed increase in waiting time post-intervention may be explained by continuing spill-over of waitlist reduction work into the post-intervention period. Reducing the waitlist took longer than anticipated, primarily due to delays in recruiting a locum neurologist who provided some additional clinics. During the final months of the implementation period, these long waiters from the original waitlist received appointments, corresponding with the increase in waiting time in the implementation period seen in Fig. 3. Some long-waiting patients from the original pre-intervention waitlist were still being allocated appointments in the first month or two of the post-intervention period, potentially inflating the median waiting time observed for new patients attending the clinic post-intervention.

A further consideration in interpreting the finding that waiting time was unchanged or increased was that after implementing STAT, *all* patients were allocated an appointment and virtually no referral was held on a waitlist. In the pre-intervention period, waiting time data were only available for patients who received an appointment. Therefore, pre-intervention, there were many patients on the waitlist who would never receive an appointment and could not be accounted for in waiting time data. This highlights the difficulty in interpreting the dual, interacting outcome measures (waitlist number and waiting time) in health services research that evaluates the impact of interventions designed to reduce waiting [31]. While patient outcomes were not evaluated in this study, STAT resulted in a fundamental change where all patients requiring the service were seen with reduced variability in waiting time and a median waiting time of six weeks from the date of referral. Consistent with guideline-directed care [1] and evidence from a systematic review [22] ensuring all patients are seen without lengthy delays could also be expected to result in improved patient outcomes.

A concern raised before implementation of the STAT model in the epilepsy clinic was that the problem of waiting might be moved to review appointments or that workload would increase. It was therefore reassuring that service stability was observed in measures such as time until the next available follow-up appointment, and number of appointments provided each week. The neurologists discharged more patients after the STAT model was implemented, perhaps due to increased confidence that, without a waitlist, their patients could re-enter the service in a timely manner if a re-referral was required. With increased awareness of data relating to service supply and demand, they became more active about triaging the limited resources they had available for ongoing management of their caseload.

Limitations of this study include the use of an historically controlled design and issues with power related to the number of data points in the interrupted time series analyses. The number of patients on the waitlist pre-intervention were only available for five of the monthly time points; missing data were managed by imputation. The post-intervention period of six months was also relatively short to evaluate the ongoing impact of implementing the STAT model. While this length of follow-up has been used in similar studies [32], more data points would have improved power and insights into whether the waitlist remained minimal and whether waiting time would change beyond this time [26]. The Quasipoisson model carried some risk of overestimating the effect due to observed overdispersion; ARIMA modelling would have been an alternative approach to address this, but the number of data points available were below the recommended quantity for this model. However, given there was no statistical change in the slope of the analysis of waiting time data, bias in overestimation of the effect is not a concern in the interpretation of the findings.

The impact of COVID-19 on this clinic was not as great as might have been expected. A study performed in this clinic prior to the pandemic to assess the feasibility of telehealth [33] meant the clinic doctors were accustomed to delivering care by telehealth. However, in a minor departure from protocol, we did not report the number of follow-up appointments as a secondary outcome [22]. There was a large increase in the number of review appointments recorded that did not reflect a true change in the quantity of service provided; changes to billing practices to accommodate telehealth in response to COVID-19 enabled previously informal phone calls (conveying information such as test results) to be formalised as review appointments.

The reduction in missed appointments post-intervention may have been achieved through stricter adherence to the existing policy informing patients they could be discharged after one missed appointment. Auditing highlighted that the chance of a patient attending a second offered appointment after one failure to attend was low and so neurologists were encouraged to discharge with an invitation to re-refer (opt-in) if required. Increased attendance may also have been influenced by more use of telehealth in the post-intervention group thereby improving access to the service [33].

The extension of the implementation period also represents a minor departure from the published protocol [22]. Given the potential for confounding that would result from incorporating the delayed backlog reduction clinics in the post-intervention period, we extended the implementation period until almost the entire backlog cohort had been offered a first appointment. Thus, an important learning for those planning to implement the model is the need to allow sufficient time for implementation.

The strength of this study is the combination of descriptive pre-post comparisons with interrupted time series analyses on a sample of almost 1000 patients. This study tackles the complex task of evaluating an intervention designed to address the enormous problem of access to outpatient services that hinders timely care for people worldwide [2, 32]. Further research is warranted in evaluating the process of implementing STAT in specialist medical outpatients and in applying this model to other specialties, or at a greater scale.

## Conclusion

The STAT model was implemented in a specialist medical epilepsy outpatient service and resulted in near-elimination of a large waitlist, and no change or a small increase in median waiting time. A reduction in variability in waiting time was observed post-intervention. This study demonstrates that medical outpatient services need not operate with lengthy waitlists if an effort is made to reduce existing backlog, combined with protection of the number of new appointments to match the rate of referral. The STAT model provides a framework for an alternative way to operate outpatient clinics that can help to ensure that all people referred are offered a timely appointment.

## Data Availability

The datasets used and analysed during the current study are available from the corresponding author on reasonable request.
